# A conformationally adaptive macrocycle: conformational complexity and host–guest chemistry of zorb[4]arene

**DOI:** 10.3762/bjoc.14.134

**Published:** 2018-06-27

**Authors:** Liu-Pan Yang, Song-Bo Lu, Arto Valkonen, Fangfang Pan, Kari Rissanen, Wei Jiang

**Affiliations:** 1 Academy of Advanced Interdisciplinary Studies, Southern University of Science and Technology, Xueyuan Blvd 1088, Shenzhen, 518055, China; 2 Department of Chemistry, Southern University of Science and Technology, Xueyuan Blvd 1088, Shenzhen, 518055, China; 3 School of Chemistry and Chemical Engineering, Harbin Institute of Technology, Harbin, 150001, China; 4 University of Jyvaskyla, Department of Chemistry and Nanoscience Center, P. O. Box 35, FI-40014, Jyvaskyla, Finland; 5 College of Chemistry, Central China Normal University, Wuhan, 430079, China

**Keywords:** conformations, host–guest chemistry, macrocycles, supramolecular chemistry, zorb[4]arene

## Abstract

Large amplitude conformational change is one of the features of biomolecular recognition and is also the basis for allosteric effects and signal transduction in functional biological systems. However, synthetic receptors with controllable conformational changes are rare. In this article, we present a thorough study on the host–guest chemistry of a conformationally adaptive macrocycle, namely per-*O*-ethoxyzorb[4]arene (**ZB4**). Similar to per-*O*-ethoxyoxatub[4]arene, **ZB4** is capable of accommodating a wide range of organic cations. However, **ZB4** does not show large amplitude conformational responses to the electronic substituents on the guests. Instead of a linear free-energy relationship, **ZB4** follows a parabolic free-energy relationship. This is explained by invoking the influence of secondary C–H···O hydrogen bonds on the primary cation···π interactions based on the information obtained from four representative crystal structures. In addition, heat capacity changes (Δ*C*_p_) and enthalpy–entropy compensation phenomena both indicate that solvent reorganization is also involved during the binding. This research further deepens our understanding on the binding behavior of **ZB4** and lays the basis for the construction of stimuli-responsive materials with **ZB4** as a major component.

## Introduction

Macrocyclic receptors are the principal workhorses used in supramolecular chemistry [1]. A myriad of synthetic macrocycles have sprouted during the past decade, greatly enriching the arsenal of supramolecular chemists [2–11]. The majority of artificial macrocycles are featured with rigid backbones as it is widely accepted that preorganization [12] is crucial for minimizing the entropy cost in molecular recognition. In contrast, bioreceptors often possess flexible backbone structures and even undergo large amplitude conformational changes upon binding substrates [13–14]. This conformational adaptivity is the basis of the allosteric effects [15–16] and signal transduction [17] observed with bioreceptors. However, similar conformationally adaptive synthetic macrocyclic receptors are relatively rare in the literature [18–23].

**Scheme 1:**
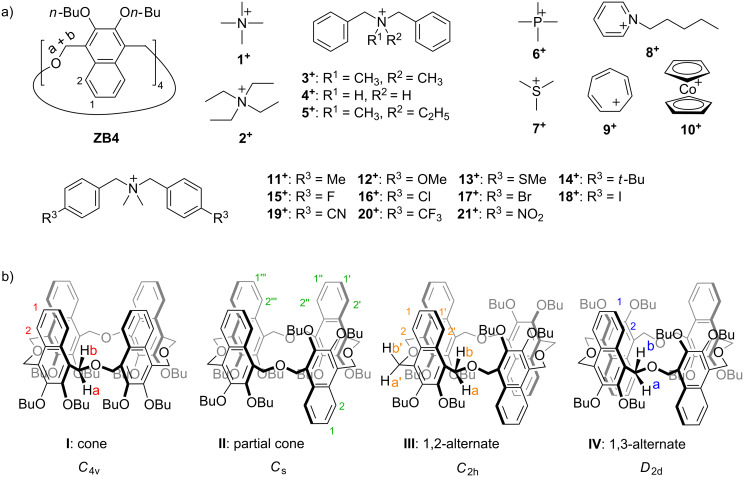
(a) Chemical structures of **ZB4** and the guests involved in this research. The counterions are PF_6_^−^. (b) The four representative conformers of **ZB4** resulting from naphthalene flipping. Numberings on the structures are used to assign NMR signals.

During the last five years, we have developed two classes of macrocyclic receptors with biomimetic structures [24]: *endo*-functionalized molecular tubes [25–30] and conformationally adaptive macrocycles [31–37]. Among the conformationally adaptive macrocycles two types were reported: oxatub[*n*]arenes [31–36] and zorb[*n*]arenes [37]. These macrocycles possess multiple conformers due to the naphthalene flipping in analogy with the phenyl-ring flipping seen in the more common calixarenes. The conformers so formed undergo quick interconversion and each one has a slightly different cavity. Thus, these conformers consist of a complex conformational network. We have carefully looked into the properties of oxatub[*n*]arenes and found that the macrocycles have many unique properties. For example, oxatub[4]arene has a wide guest scope and can bind almost all of the common organic cations [32]. It also shows conformational responses to solvent change [33] and remote electronic substituents on the guests [34]. In addition, different alkyl side chains on oxatub[4]arenes lead to different macroscopic self-assembly behaviors [36]. Zorb[4]arene was first synthesized, reported and so named by the Georghiou group in 2005. The derivatives per-*O*-methoxy- and per-*O*-ethoxyzorb[4]arene were shown to be effective tetramethylammonium ion receptors [38]. The per-*O*-*n*-butoxyzorb[4]arene (**ZB4**, Scheme 1a) was only recently further studied by us with respect to its rich conformational properties and the consequence on macroscopic self-assembly [37]. In the present research, we report the binding behavior of **ZB4** to a much wider guest scope. We found that the guest-binding ability and conformational adaptivity of **ZB4** are quite different from that of per-*O*-*n*-butoxyoxatub[4]arene (**TA4**).

## Results and Discussion

Conformational adaptability enables oxatub[4]arenes to host a wide range of organic cations [32]. **ZB4** is also a conformationally adaptive macrocycle. We wondered whether **ZB4** has a wide guest binding scope. It was reported that quaternary ammonium-based organic cations (**1^+^**–**3^+^**) can be hosted by zorb[4]arenes [37–38]. Quaternary ammonium cations **4^+^** and **5^+^** and other types of organic cations hosted by **TA4** (**6^+^**–**10^+^**) were tested with **ZB4**. Most of these guests can indeed be complexed. But there are some exceptions. Changing the core quaternary ammonium structure of **3^+^** completely shuts down the binding, because no obvious complexation-induced shifts were detected in the 1:1 mixture of **ZB4** with **4^+^** or **5^+^** (Figures S1 and S2 in Supporting Information File 1). This indicates the importance of the core quaternary ammonium ions in the host−guest complexation. All other guests can be encapsulated in the cavity of **ZB4**, and significant chemical shifts on both the guests and **ZB4** were observed in the NMR spectra (Figures S3–S7 in Supporting Information File 1). The ESI mass spectra of equimolar mixtures of guests **9^+^** and **10^+^** and **ZB4** were obtained (Figures S9 and S10 in Supporting Information File 1) and the predominant peaks were assigned to 1:1 complexes after losing PF_6_^−^.

NMR titrations and isothermal titration microcalorimetry (ITC) were then performed to obtain the association constants. For small guests such as **1^+^**, **2^+^**, **6^+^**–**9^+^**, NMR titration experiments with **ZB4** have been performed due to the fast equilibrium of the free and **ZB4**-complexed guests on the NMR time scale. All titration curves agreed well with a 1:1 stoichiometry (Figures S11–S14 in Supporting Information File 1). In case of guests **3^+^** and **10^+^**, the binding heats were high enough to be measured. Thus the binding parameters were determined by ITC titrations (Figure S15 in Supporting Information File 1) and the results are shown in Table 1. Generally, **ZB4** shows weaker binding affinities to these guests than **TA4** does with the same counterions. For example, **ZB4** and cation **3^+^**, with a binding constant of 5.4 × 10^4^ M^−1^, was the best guest among the studied ones. However, the corresponding association constant with **TA4** has been 1.7 × 10^5^ M^−1^. Similar differences were also observed for cations **9^+^** and **10^+^**, their binding affinities with **ZB4** were lower by 1–2 orders of magnitude than those with **TA4**. However, the small guests, such as **2^+^** and **6^+^**–**8^+^** share rather similar binding affinities to both **ZB4** and **TA4**.

**Table 1:** Association constants (M^−1^) and other thermodynamic parameters as determined by ^1^H NMR titrations (400 MHz, CD_2_Cl_2_/CD_3_CN 1:1, 298 K) or by ITC titrations in a 1:1 mixture of 1,2-dichloroethane and MeCN at 298 K.

guests^a^	*K*_a_ (M^−1^)		guests^a^	*K*_a_ (M^−1^)

**1^+^**^b^	4700 ± 600		**7^+^**	349 ± 29
**2^+^**^b^	590 ± 30		**8^+^**	468 ± 31
**6^+^**	524 ± 48		**9^+^**	1300 ± 100

guests^c^	*K*_a_ (M^−1^)	Δ*G* (kJ∙mol^−1^)	Δ*H* (kJ∙mol^−1^)	−*T*Δ*S* (kJ∙mol^−1^)

**3^+^**^b^	(5.4 ± 1.2) × 10^4^	−27.0 ± 0.8	−31.6	4.6
**10^+^**	(4.3 ± 1.0) × 10^4^	−26.5 ± 0.7	−18.1	−8.4

^a^The association constants were determined by NMR titrations; ^b^the binding parameters of these guests have been reported (see ref. [37]); ^c^the association constants were determined by ITC titrations.

**Figure 1:**
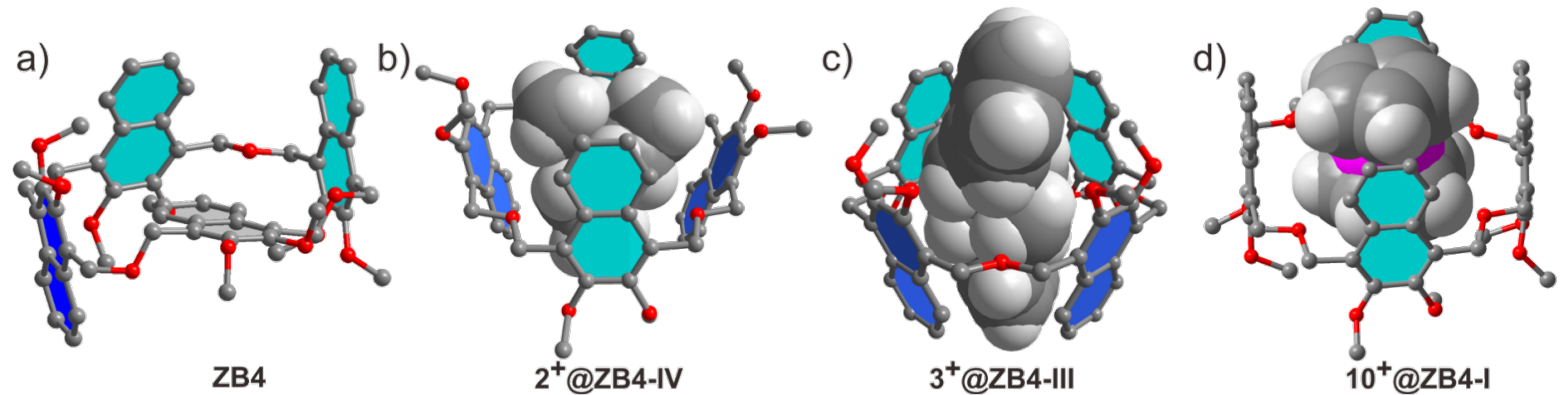
X-ray single crystal structure of **ZB4** and the host–guest complexes. a) **ZB4**, b) **2^+^**@**ZB4-IV,** c) **3^+^**@**ZB4-IV,** d) **10^+^**@**ZB4-I**. Hydrogen atoms of the host are removed and butyl groups are shortened to methyl groups for viewing clarity. The X-ray single crystal structures of **2^+^**@**ZB4-IV** (b) and **3^+^**@**ZB4-III** (c) have been reported previously (see [37]).

The X-ray crystal structure of free **ZB4** shows it to exist as a self-inclusion conformation in the solid state (Figure 1a). This conformation is different from the ones containing different lower-rim alkyl groups reported earlier [37–38]. Crystals were obtained by slow evaporation of the compounds’ CH_3_CN solutions and the different conformations in the solid state may result from the packing of the different lower-rim alkyl groups. For the conformers with cavities (Scheme 1b), three out of the four have been predominantly selected by three different guests. For example, guests **2^+^** and **3^+^** induced conformers **IV** and **III**, respectively, to achieve optimal binding [37]. This has been unambiguously confirmed by X-ray single crystallography (Figure 1b and 1c). Guest **10^+^** is a strong binder and its induction on the conformations of **ZB4** was further analyzed. The guest exchange in solution of **10^+^**@**ZB4** is fast/intermediate on the NMR timescale, as witnessed by broadening of all signals in the spectrum at 25 °C (Figure S7b in Supporting Information File 1). Thus, a ^1^H NMR experiment at −20 °C was performed to slow down the guest exchange. Indeed, the protons a and b are clearly separated, suggesting that the guest exchange is now slow on the NMR timescale (Figure S7c in Supporting Information File 1). Only two signals for the aromatic protons of the host are observed, suggesting that **ZB4** predominantly exists as either conformer **I** or **IV** in the complex **10^+^**@**ZB4**. However, it has been not clear which one **ZB4** adopts. Fortunately, a single crystal suitable for X-ray diffraction could be obtained by slow evaporation of the solution of **10^+^** and **ZB4** in a mixture of CH_2_Cl_2_ and CH_3_CN. The crystal structure clearly shows that conformer **I** (Figure 1d) is the selected conformation by guest **10^+^**.

**TA4** shows a large amplitude of conformational change in response to the remote electronic substituents on the guests [34]. We wondered whether a similar behavior would be observed for **ZB4**. Consequently, a series of guests with different substituents in the *para*-positions of guest **3^+^** were employed to study the electronic substituent effect of the guests on the binding behavior of **ZB4**. As the guest exchange is slow on the NMR timescale the experiments were performed by ^1^H NMR spectroscopy. Surprisingly, all the ^1^H NMR spectra of the complexes (Figure S8 in Supporting Information File 1) shared similar peak patterns as **3^+^**@**ZB4**, suggesting conformer **III** (*C*_2h_ symmetry) [37] to be the most favored conformation for all complexes. Obviously the conformational network of **ZB4** shows no response to the electronic substituents on the guests. This is quite different from **TA4**.

**Figure 2:**
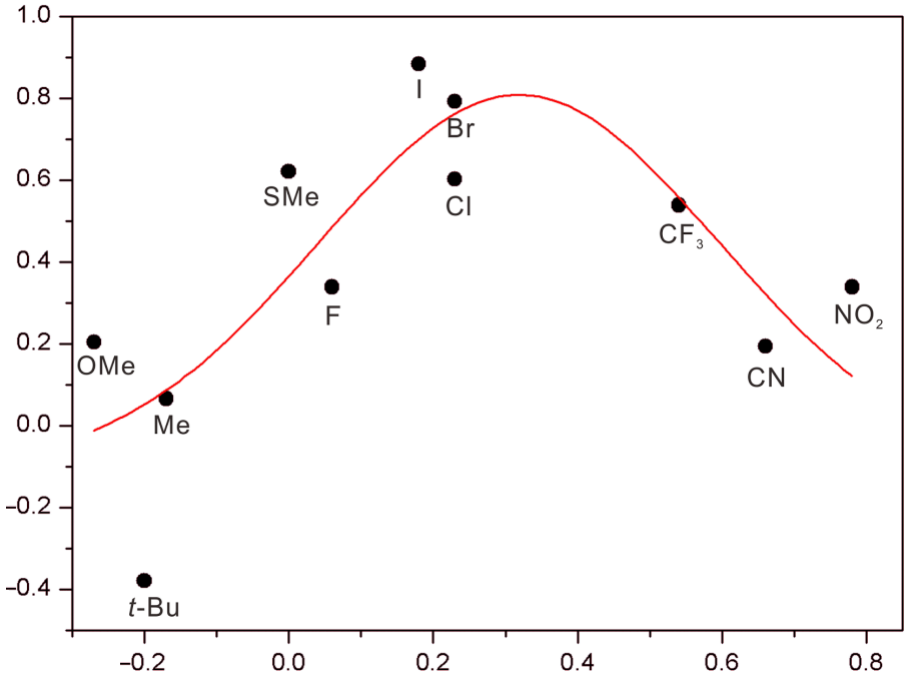
Parabolic free-energy relationship between log(*K*_R_/*K*_H_) and Hammett parameter σ_p_. *K*_R_: guests **11^+^**–**21^+^**; *K*_H_: guest **3^+^**.

In addition, there has been a linear free energy relationship between electronic properties of substituents present in the guests and their binding affinities with **TA4**, indicating that the binding affinities are affected by substituents through a field/inductive effect [34]. However, this is again quite different for **ZB4**. The association constants of **ZB4** to these guests were determined by ITC titrations (Figures S15–S26 in Supporting Information File 1), and the data are shown in Table 2. The logarithm of the corresponding association constants of **11^+^**–**21^+^** over **3^+^** were parabolic as the function of Hammett parameter (σ_p_) [39] (Figure 2). For substituents C(Me)_3_, OMe, Me, SMe, F, Cl, Br, and I, the binding affinities increase with increasing σ_p_. However, the binding affinities decrease with further increasing σ_p_ (CF_3_, NO_2_, and CN). The guest with iodo substituent (**18^+^**) is the best, with an association constant of 4.9 × 10^5^ M^−1^ at 25 °C. It is interesting to note that although the substituents of guests **12^+^** and **19^+^** are quite different in view of their electronic properties, they share very similar binding affinities.

**Table 2:** Association constants (M^−1^) and other thermodynamic parameters of **ZB4** with guests **11^+^**–**21^+^** as determined by ITC titrations in a 1:1 mixture of 1,2-dichloroethane and MeCN at 298 K.

guests	R^3^	*K*_a_	Δ*G* (kJ∙mol^−1^)	Δ*H* (kJ∙mol^−1^)	−*T*Δ*S* (kJ∙mol^−1^)

**11^+^**	CH_3_	(6.4 ± 0.5) × 10^4^	−27.4 ± 0.5	−37.0	9.6
**12^+^**	OMe	(8.8 ± 1.1) × 10^4^	−28.2 ± 0.7	−38.1	9.8
**13^+^**	SMe	(2.3 ± 0.2) × 10^5^	−30.6 ± 0.7	−50.1	19.5
**14^+^**	*t*-Bu	(2.3 ± 0.6) × 10^4^	−24.9 ± 0.5	−40.7	15.8
**15^+^**	F	(1.2 ± 0.1) × 10^5^	−29.0 ± 0.7	−35.6	6.6
**16^+^**	Cl	(2.2 ± 0.3) × 10^5^	−30.6 ± 0.8	−37.3	6.7
**17^+^**	Br	(3.4 ± 0.2) × 10^5^	−31.5 ± 0.8	−39.0	7.5
**18^+^**	I	(4.9 ± 0.7) × 10^5^	−32.5 ± 0.9	−40.0	7.5
**19^+^**	CN	(8.6 ± 1.5) × 10^4^	−28.2 ± 0.7	−32.4	4.2
**20^+^**	CF_3_	(1.9 ± 0.2) × 10^5^	−30.1 ± 0.9	−37.4	7.3
**21^+^**	NO_2_	(1.2 ± 0.1) × 10^5^	−30.4 ± 0.5	−32.6	3.2

**Figure 3:**
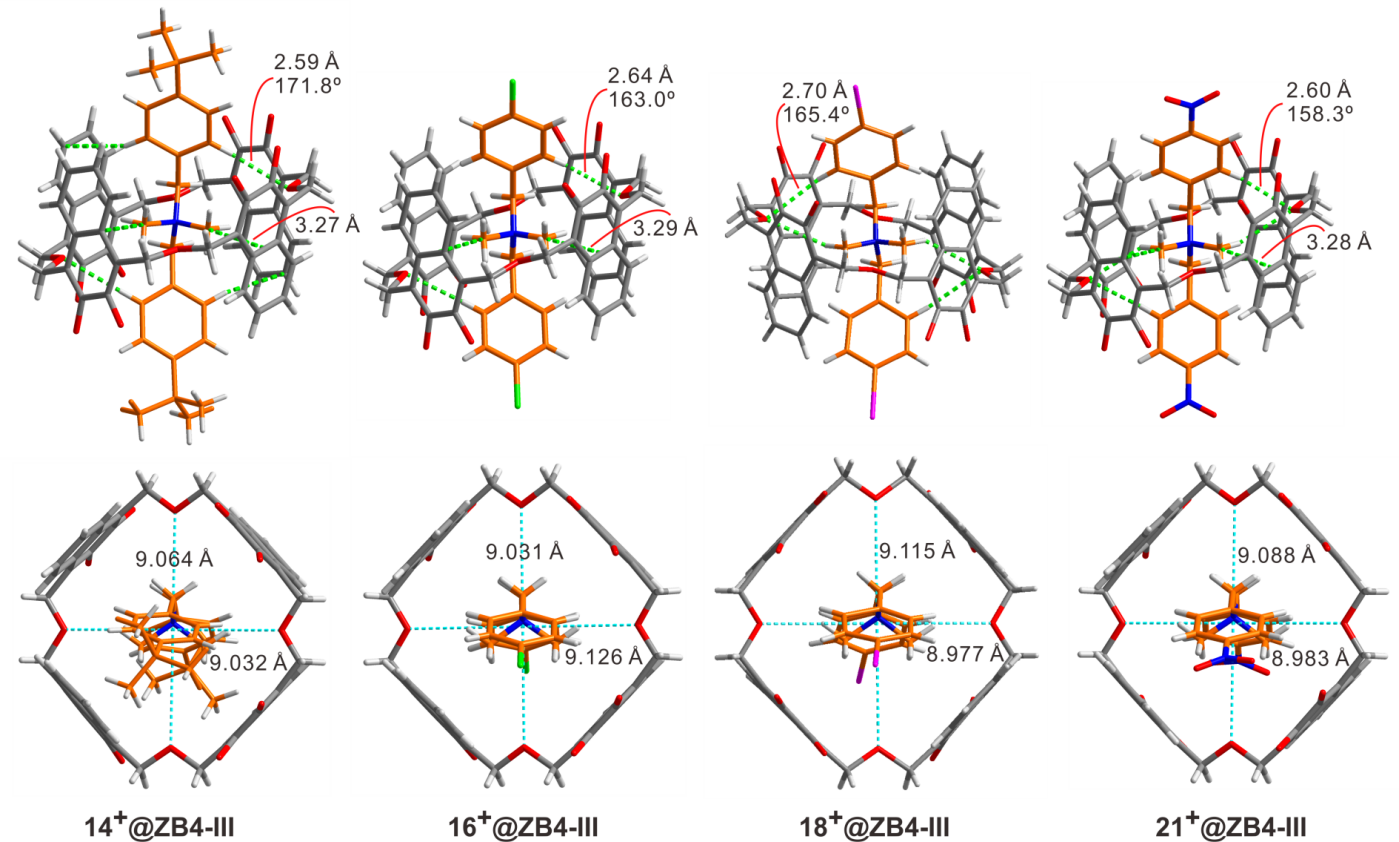
X-ray single crystal structures of **14^+^**@**ZB4-III**, **16^+^**@**ZB4-III**, **18^+^**@**ZB4-III** and **21^+^**@**ZB4-III**. Butyl groups of the host are removed for viewing clarity.

**Figure 4:**
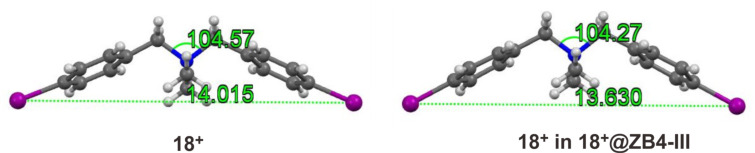
X-ray single crystal structures of **18^+^@ZB4-III** and **18^+^** in **18^+^@ZB4-III**.

What is the underlying reason for the response of binding affinities to the electronic substituent effect on the guests? Luckily, single crystals of complexes **14^+^**@**ZB4-III**, **16^+^**@**ZB4-III**, **18^+^**@**ZB4-III**, and **21^+^**@**ZB4-III** suitable for X-ray single crystallography, were obtained and their crystal structures are shown in Figure 3. The substituents of these four guests located at three representative positions in Figure 2. Therefore, a closer look at their crystal structures may provide an explanation for their surprising binding behaviors. Multiple non-covalent interactions, including C–H···O hydrogen bonds, cation···π, C–H···π and π···π interactions, are involved in all the cases. Undoubtedly, cation···π interactions between the core quaternary ammonium ions of the guests and the four naphthalene rings of the host should still be the major driving force as mentioned above. However, it was noticed that the distances between diagonal linker oxygen atoms in the backbone of the host are slightly different for the four complexes. These interactions may be tuned by the size of the host cavity. As shown in Figure 3 (bottom), the vertical and horizontal distances between the diagonal oxygen atoms are different for all the four complexes. This distance is presumably tuned through the C–H···O hydrogen bonds between the CH_2_–O–CH_2_ oxygen atoms and the aromatic protons of the guests. The acidities of aromatic protons on the guests are, however, influenced by the substituents. Indeed, the electron-withdrawing nitro group and the electron-donating *tert*-butyl group both result in shorter C–H···O hydrogen bonds than the chloro and iodo groups do. The cavity sizes of **ZB4** in complexes **16^+^**@**ZB4-III** and **18^+^**@**ZB4-III** may be better suited than those of **14^+^**@**ZB4-III** and **21^+^**@**ZB4-III** to host the quaternary ammonium and maximize all the non-covalent interactions. Any deviation from these cavity sizes weakens the binding. That is, the secondary C–H···O hydrogen bonds can be tuned through the substituents to leverage the primary cation···π interactions and thus the final binding affinities. This may explain the parabolic distribution of binding affinities over the Hammett parameters (σ_p_) of the substituents as shown in Figure 2. The conformational adaptivity or flexibility allows **ZB4** to adapt according to the need of the guests. Simultaneously, the guest may also conformationally adapt to better interact with **ZB4**. As shown in Figure 4, the crystal structure of **18^+^** in **18^+^**@**ZB4-III** is slightly different in shape from free cation **18^+^**.

**Figure 5:**
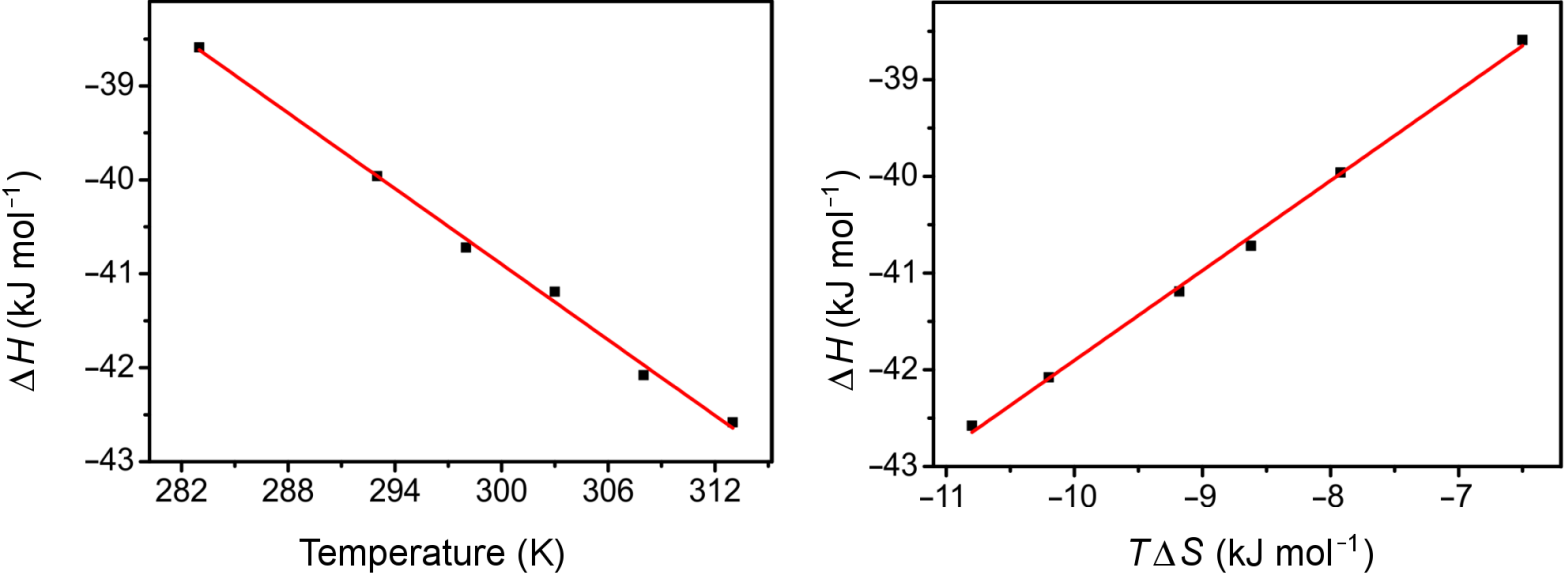
Linear relationships of Δ*H* with temperature (left, slope = −0.13, R^2^ = 0.9956) and *T*Δ*S* (right, slope = 0.93, R^2^ = 0.9976).

In addition, thermodynamic parameters at different temperatures for the complex between **ZB4** and **18^+^** were determined by ITC experiments (Figure S27 in Supporting Information File 1). The data are compiled in Table 3. The heat capacity change (Δ*C*_p_) for the formation of **18^+^**@**ZB4** is −0.13 kJ mol^−1^ K^−1^ as determined from the slope of the linear fitting in plots of Δ*H* versus temperature from 283 to 313 K (Figure 5). The release of solvent molecules upon complex formation may account for the negative heat capacity change and similar heat capacity changes were also reported for the fullerenes recognition [40]. Meanwhile, the changes of Δ*G* for the formation of **18^+^**@**ZB4** complex over the temperature range 283–313 K is very small (0.31 kJ mol^−1^), while the changes in Δ*H* and −*T*Δ*S* are much larger (ca. 4–4.3 kJ mol^−1^). The changes in Δ*H* and −*T*Δ*S* are opposite in signs and perfectly compensate each other. The enthalpy–entropy compensation phenomenon may be explained by invoking a solvent reorganization during the formation of **18^+^**@**ZB4** complex, which is common for reactions taking place in aqueous solution [41].

**Table 3:** Thermodynamic parameters for the complex between **18^+^** and **ZB4** as determined by ITC in a 1:1 mixture of 1,2-dichloroethane and MeCN at different temperatures.

*T* (K)	*K*_a_ (M^−1^)	Δ*G* (kJ∙mol^−1^)	Δ*H* (kJ∙mol^−1^)	−*T*Δ*S* (kJ∙mol^−1^)

283	8.4 × 10^5^	−32.09	−38.59	6.50
293	5.2 × 10^5^	−32.04	−39.96	7.92
298	4.2 × 10^5^	−32.10	−40.72	8.62
303	3.3 × 10^5^	−32.01	−41.19	9.18
308	2.6 × 10^5^	−31.88	−42.08	10.2
313	2.0 × 10^5^	−31.78	−42.58	10.8

## Conclusion

In summary, we systematically studied the guest binding scope, electronic substituent effects and thermodynamic origin on the molecular recognition of **ZB4** using NMR, ITC titration and X-ray crystallography. Similar to **TA4**, **ZB4** is able to host a wide range of organic cations. However, in contrast to **TA4**, **ZB4** shows no large amplitude of conformational response to the electronic nature of substituents on the guests, and its binding affinities follow a parabolic rather than a linear free energy relationship. A closer look at four representative crystal structures suggested that the parabolic free energy relationship may be caused through influencing the major interactions in the host–guest complexes by tuning the weak C–H∙∙∙O hydrogen bonds. Heat capacity changes and enthalpy–entropy compensation indicate that solvent reorganization is also involved during the host–guest binding. Generally, **ZB4** is quite different from **TA4**, and further enriches the arsenal of conformationally adaptive macrocycles. With these model systems, we may further understand the importance of conformational adaptivity in biomolecular recognition and even design stimuli-responsive materials by harnessing this large amplitude of conformational changes.

## Supporting Information

File 1 Experimental procedures, NMR spectra, mass spectra, determination of association constants and X-ray single crystal data.
