# Supplementary material for: A conformationally adaptive macrocycle: conformational complexity and host–guest chemistry of zorb[4]arene
Source: Beilstein J Org Chem. 2018 Jun 27;14:1570–7. doi: 10.3762/bjoc.14.134 (PMC6037019; doi:10.3762/bjoc.14.134)
Supplement: File 1 — Experimental procedures, NMR spectra, mass spectra, determination of association constants and X-ray single crystal data. [file Beilstein_J_Org_Chem-14-1570-s001.pdf]

Supporting Information

for

A conformationally adaptive macrocycle: conformational complexity and host–guest chemistry of zorb[4]arene

Liu-Pan Yang[1,3], Song-Bo Lu[2,3], Arto Valkonen[4], Fangfang Pan[5], Kari Rissanen[4] and Wei Jiang*[3]

Address: [1]Academy of Advanced Interdisciplinary Studies, Southern University of Science and Technology, Xueyuan Blvd 1088, Shenzhen, 518055, China, [2]School of Chemistry and Chemical Engineering, Harbin Institute of Technology, Harbin, 150001, China, [3]Department of Chemistry, Southern University of Science and Technology, Xueyuan Blvd 1088, Shenzhen, 518055, China, [4]University of Jyvaskyla, Department of Chemistry and Nanoscience Center, P. O. Box 35, FI-40014, Jyvaskyla, Finland and [5]College of Chemistry, Central China Normal University, Wuhan, 430079, China

Email: Wei Jiang - jiangw@sustc.edu.cn

* Corresponding author

Experimental procedures, NMR spectra, mass spectra, determination of association constants and X-ray single crystal data

Table of Contents

1. Experimental section S2
2. ^{1}H NMR spectra of the complexes S3
3. Mass spectra of host–guest complexes S8
4. Binding constants determined by NMR titration S9
5. Binding constants determined by ITC titration S11
6. X-Ray single crystal data S18
7. References S22

1. Experimental section

General methods. All the reagents involved in this research were commercially available and used without further purification unless otherwise noted. Solvents were either employed as purchased or dried prior to use by standard laboratory procedures. ^{1}H NMR spectra were recorded on Bruker Avance-400 (500) spectrometers. All chemical shifts are reported in ppm with residual solvents or TMS (tetramethylsilane) as the internal standards. Electrospray-ionization time-of-flight high-resolution mass spectrometry (ESI-TOF-HRMS) experiments were conducted on an applied biosystems Elite ESI-QqTOF mass spectrometry system.

Isothermal titration calorimetry, ITC. Titration experiments were carried out in 1,2-dichloroethane/CH_3CN 1:1 (v/v) at 298 K on a Nano ITC LV – 190 μL (Waters GmbH, TA Instruments, Eschborn, Germany). In a typical experiment, a 190 μL solution of **ZB4** was placed in the sample cell at a concentration of 0.15 mM, and 50 μL of a solution of the hexafluorophosphate salt (1 mM in the same solvent) was in the injection syringe. The titrations consisted of 25 consecutive injections of 1.96 μL each with a 200s interval between the individual injections. Heats of dilution, measured by titration of the salt into the sample cell with blank solvent, were subtracted from each data set. All solutions were degassed prior to titration. The data were analyzed using the instrumental internal software package and fitted with a 1:1 binding model. Errors are smaller than ±10%.

2. ^{1}H NMR spectra of the complexes

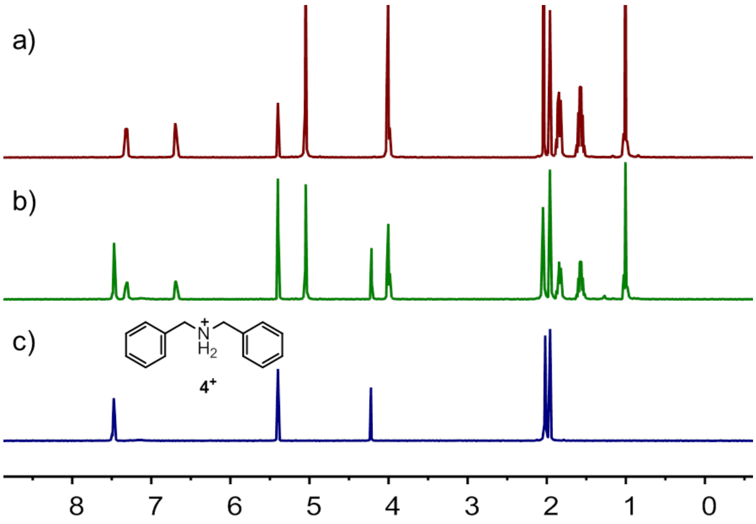

Figure S1: ^{1}H NMR spectra (400 MHz, CD_2Cl_2/CD_3CN 1:1, 2.0 mM, 298 K) of (a) **ZB4**, (c) **4-PF$_6$** and (b) their equimolar mixture. In the host–guest mixture, the protons of the guest and host undergo no obvious shift, suggesting there is no complexation between the host and the guest.

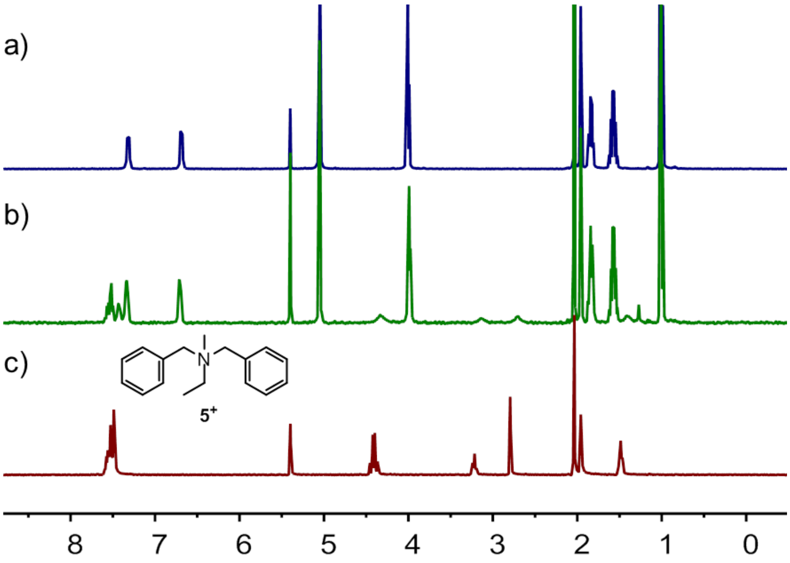

Figure S2: ^{1}H NMR spectra (400 MHz, CD_2Cl_2/CD_3CN 1:1, 2.0 mM, 298 K) of (a) **ZB4**, (c) **5-PF$_6$** and (b) their equimolar mixture. In the host–guest mixture, the protons of the guest and host undergo no obvious shift, suggesting there is no complexation between the host and the guest.

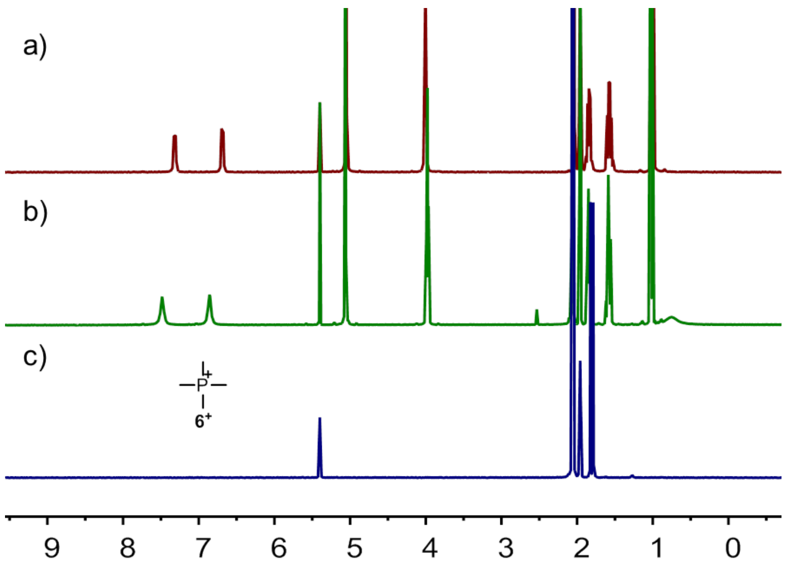

Figure S3: ^{1}H NMR spectra (400 MHz, CD_2Cl_2/CD_3CN 1:1, 2.0 mM, 298 K) of (a) **ZB4**, (c) **6-PF$_6$** and (b) their equimolar mixture. In the host–guest mixture, the protons of the guest shifted upfield, while the protons of **ZB4** underwent downfield shift, suggesting a binding event.

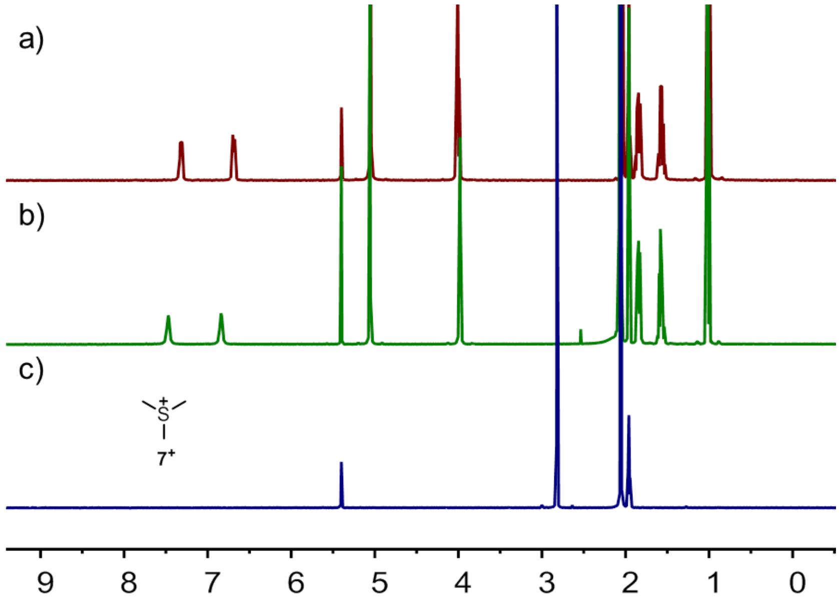

Figure S4: ^{1}H NMR spectra (400 MHz, CD_2Cl_2/CD_3CN 1:1, 2.0 mM, 298 K) of (a) **ZB4**, (c) **7-PF$_6$** and (b) their equimolar mixture. In the host–guest mixture, the protons of the guest shifted upfield, while the protons of **ZB4** underwent downfield shift, suggesting a binding event.

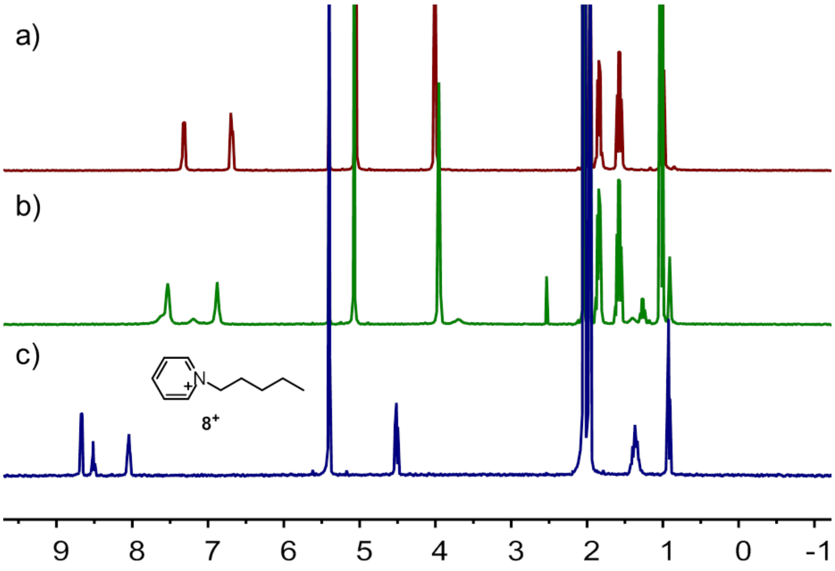

Figure S5: ^{1}H NMR spectra (400 MHz, CD_2Cl_2/CD_3CN 1:1, 2.0 mM, 298 K) of (a) **ZB4**, (c) **8-PF$_6$**, and (b) their equimolar mixture. In the host–guest mixture, the protons of the guest shifted upfield, while the protons of **ZB4** underwent downfield shift, suggesting a binding event.

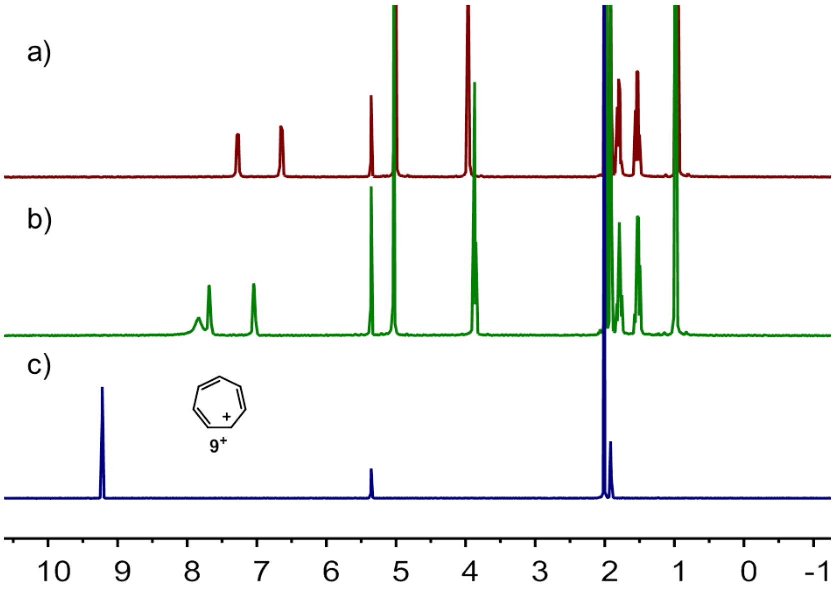

Figure S6: ^{1}H NMR spectra (400 MHz, CD_2Cl_2/CD_3CN 1:1, 2.0 mM, 298 K) of (a) **ZB4**, (c) **9-PF$_6$** and (b) their equimolar mixture. In the host–guest mixture, the protons of the guest shifted upfield, while the protons of **ZB4** underwent downfield shift, suggesting a binding event.

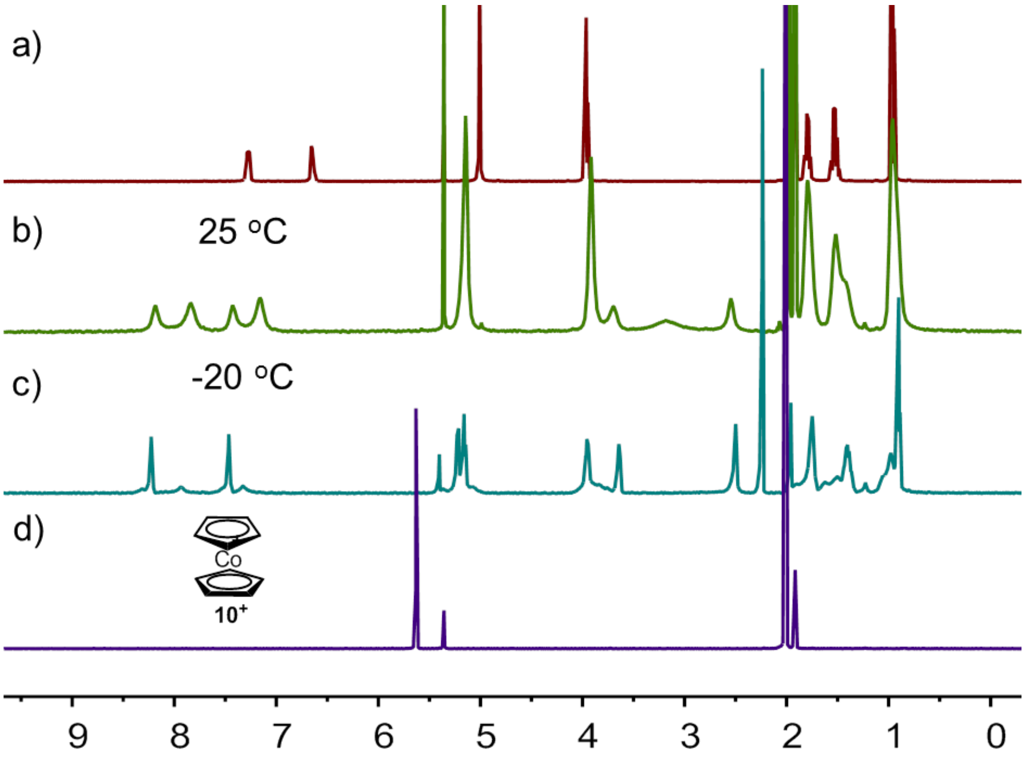

Figure S7: ^{1}H NMR spectra (400 MHz, CD_2Cl_2/CD_3CN 1:1, 2.0 mM) of (a) **ZB4**, (d) **10-PF$_6$** andtheir equimolar mixture at (b) 25 °C and (c) −20 °C. In the host–guest mixture, the protons of the guest shifted upfield, while the protons of **ZB4** underwent downfield shift, suggesting a binding event.

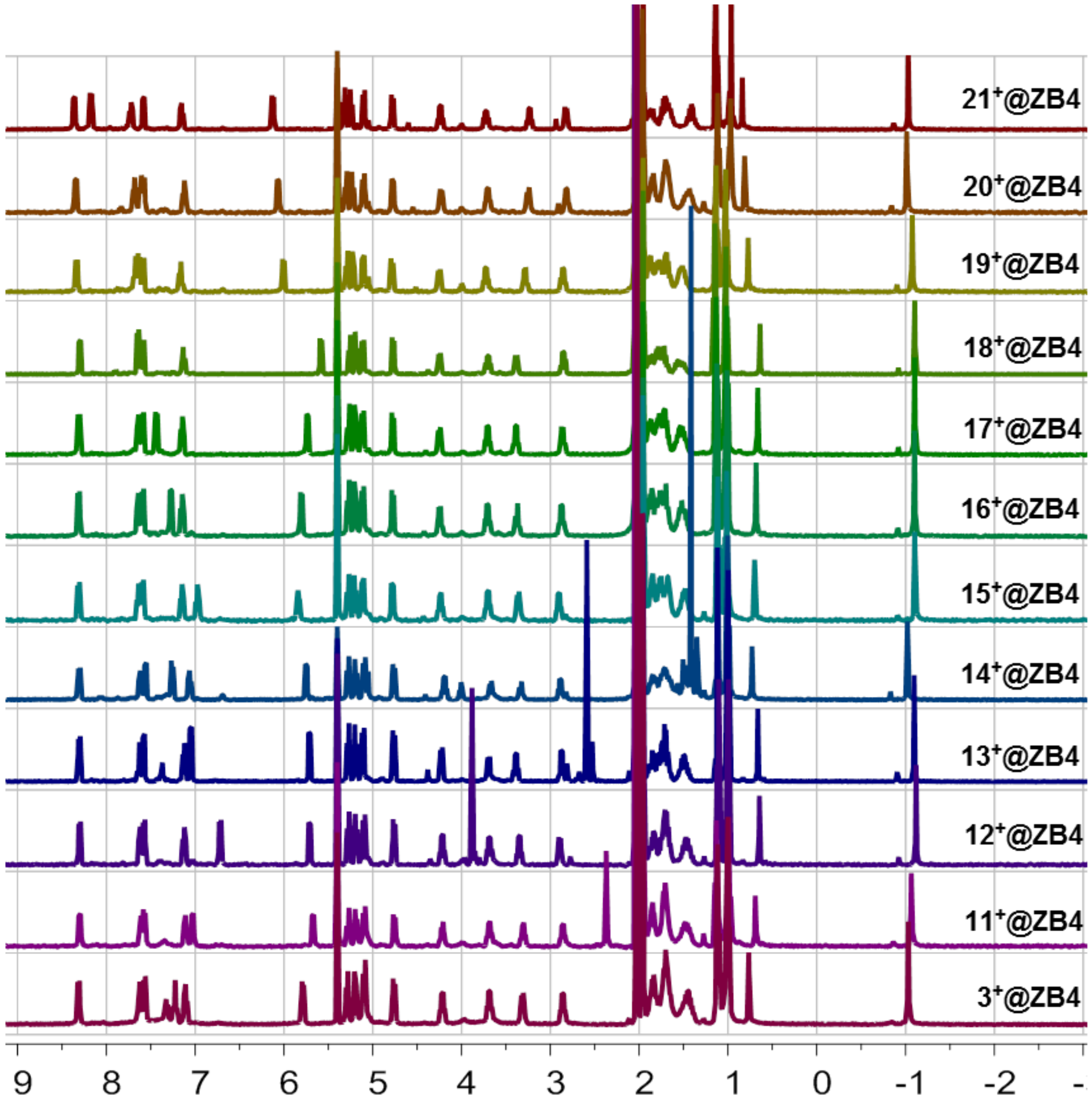

Figure S8: ^{1}H NMR spectra (400 MHz, CD_2Cl_2/CD_3CN 1:1, 2.0 mM, 298 K) of 1:1 mixtures of **ZB4** with individual guest $\mathbf{3}^{+}$ or $\mathbf{11}^{+}$–$\mathbf{21}^{+}$.

3. Mass spectra of host–guest complexes

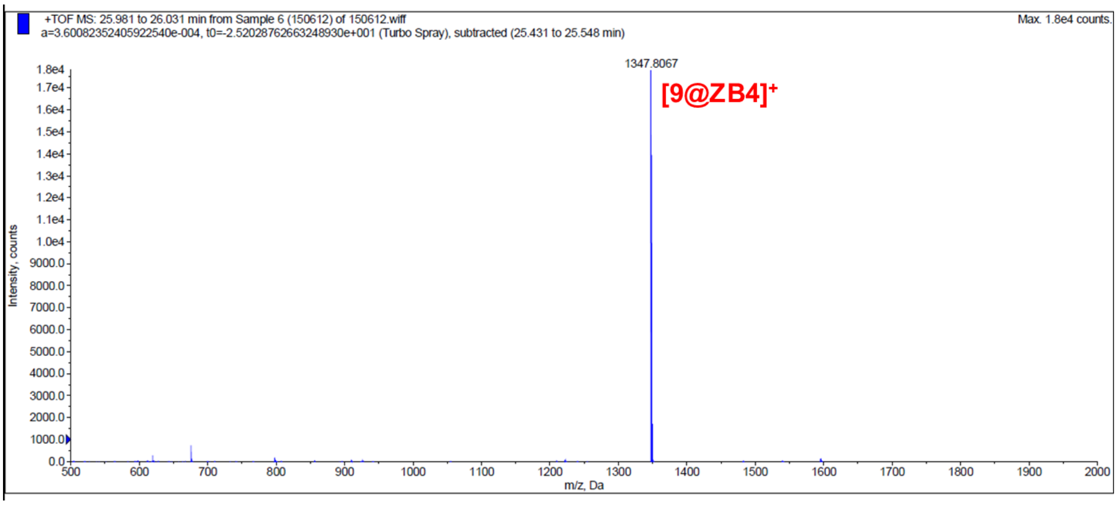

Figure S9: ESI mass spectrum of **9-2PF$_6$@ZB4**. The result indicates **9-2PF$_6$** and **ZB4** form a 1:1 complex.

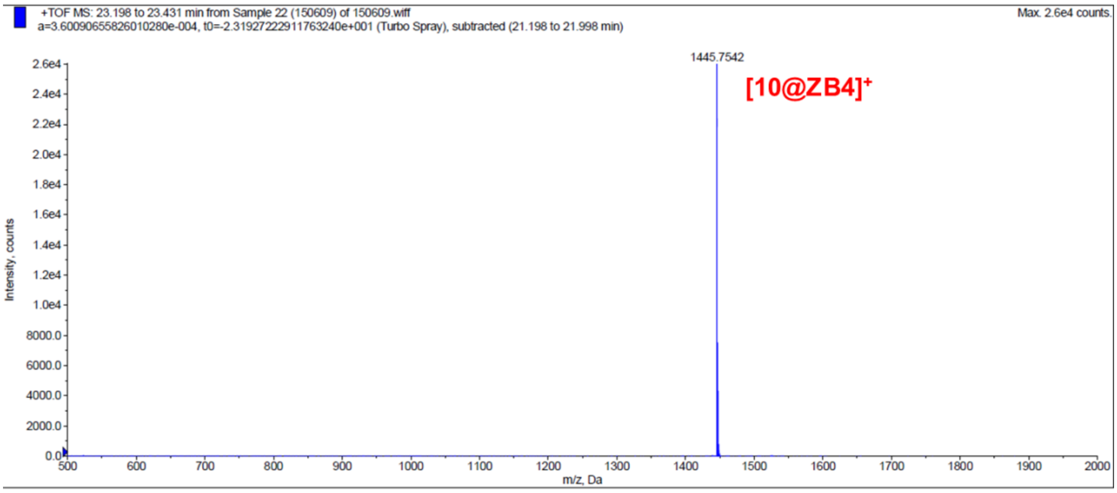

Figure S10: ESI mass spectrum of **10-2PF$_6$@ZB4**. The result indicates **10-2PF$_6$** and **ZB4** form a 1:1 complex.

4. Binding constants determined by NMR titrations

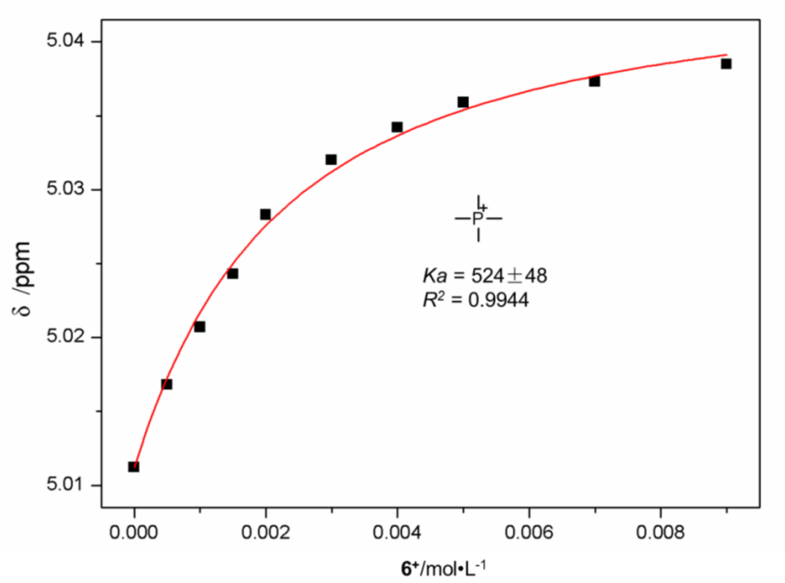

Figure S11: Non-linear curve-fitting of NMR titrations for the complexation between **ZB4** and **6-PF$_6$** in the 1:1 mixture of CD_2Cl_2 and CD_3CN at 298 K. Nonlinear curve-fitting method used here has been reported.[1]

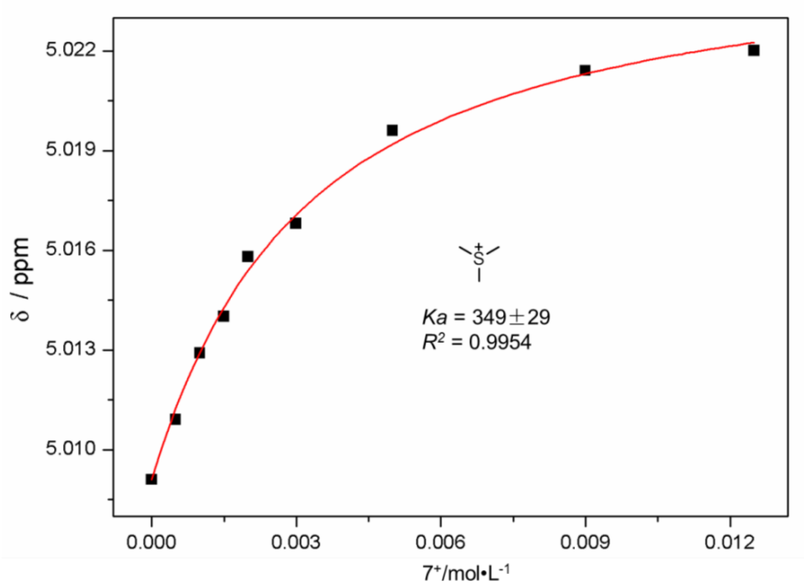

Figure S12: Non-linear curve-fitting of NMR titrations for the complexation between **ZB4** and **7-PF$_6$** in the 1:1 mixture of CD_2Cl_2 and CD_3CN at 298 K.

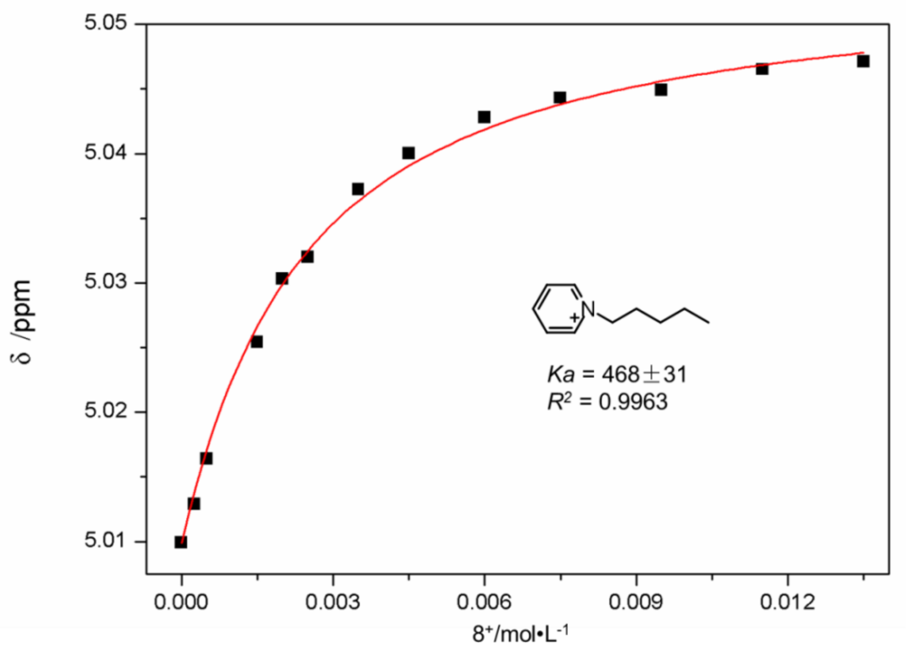

Figure S13: Non-linear curve-fitting of NMR titrations for the complexation between **ZB4** and **8-PF$_6$** in the 1:1 mixture of CD_2Cl_2 and CD_3CN at 298 K.

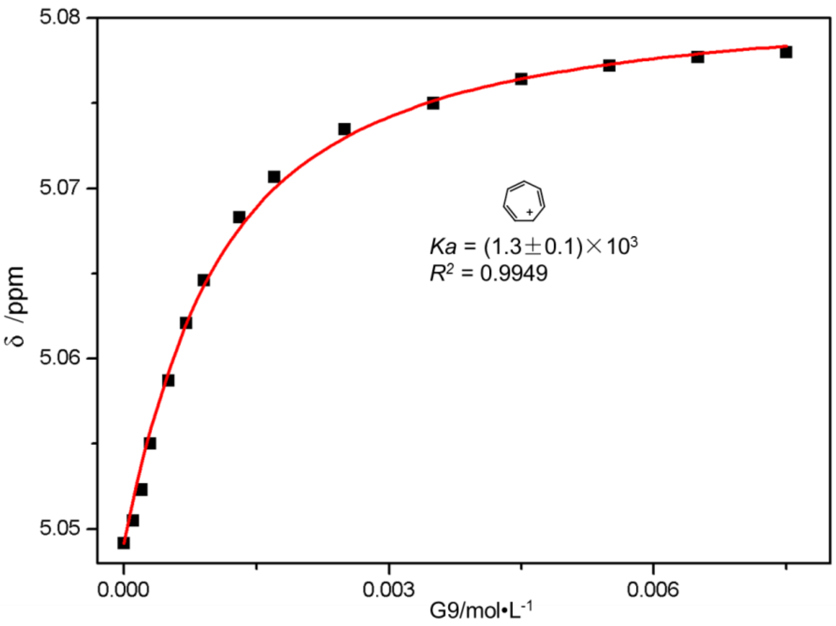

Figure S14: Non-linear curve-fitting of NMR titrations for the complexation between **ZB4** and **9-PF$_6$** in the 1:1 mixture of CD_2Cl_2 and CD_3CN at 298 K.

5. Binding constants determined by ITC titrations

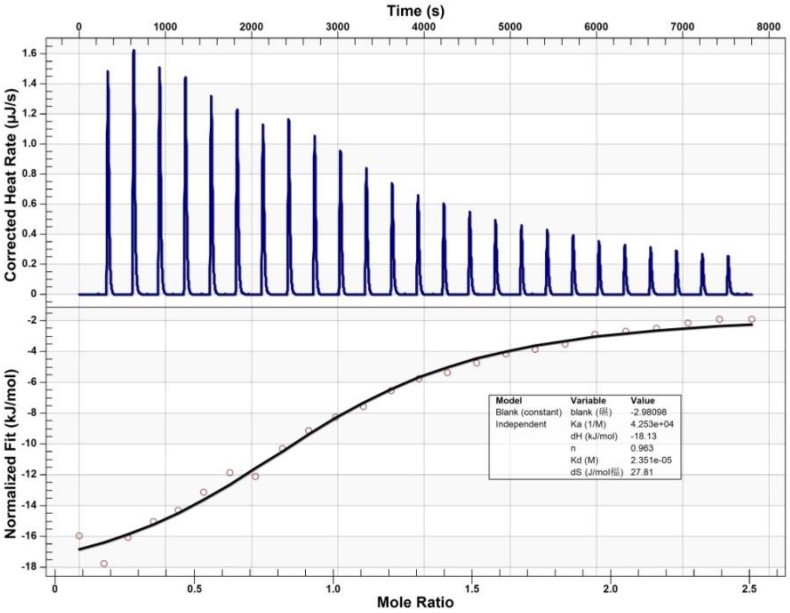

Figure S15: Titration plots (heat flow versus time and heat versus guest/host ratio) obtained from ITC experiments of **ZB4** with **10**-PF_6 in the 1:1 mixture of 1,2-dichloroethane and CH_3CN.

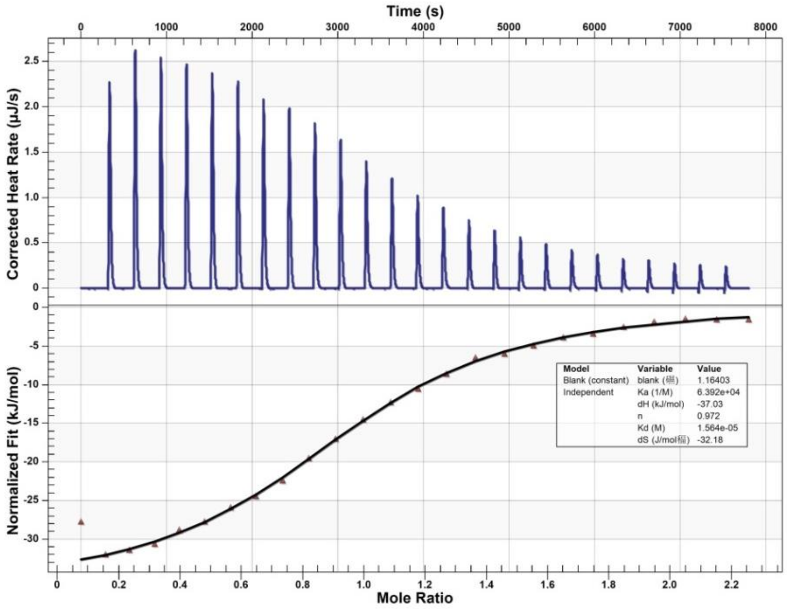

Figure S16: Titration plots (heat flow versus time and heat versus guest/host ratio) obtained from ITC experiments of **ZB4** with **11**-PF_6 in the 1:1 mixture of 1,2-dichloroethane and CH_3CN.

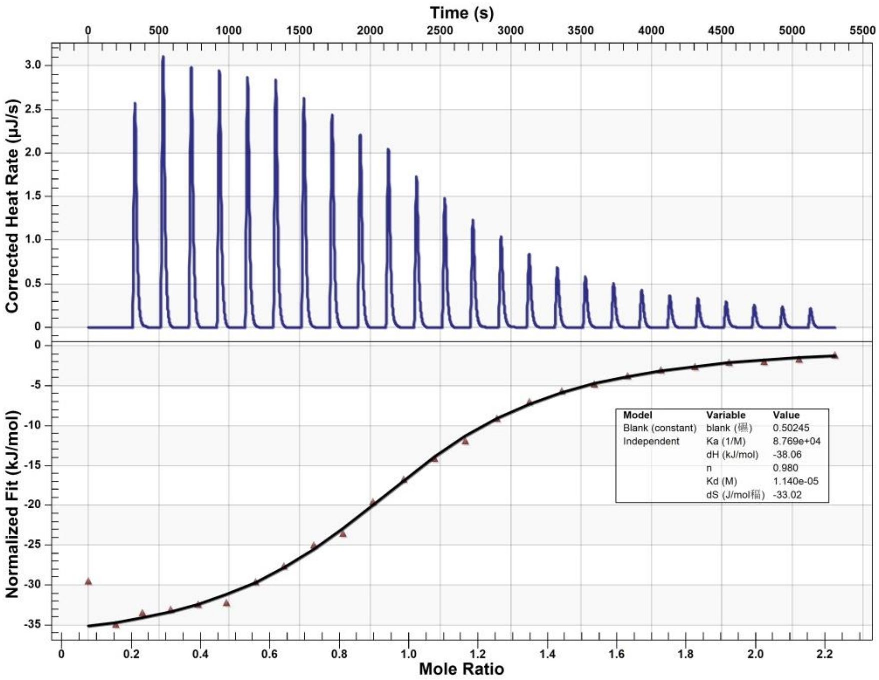

Figure S17: Titration plots (heat flow versus time and heat versus guest/host ratio) obtained from ITC experiments of **ZB4** with **12**-PF_6 in the 1:1 mixture of 1,2-dichloroethane and CH_3CN.

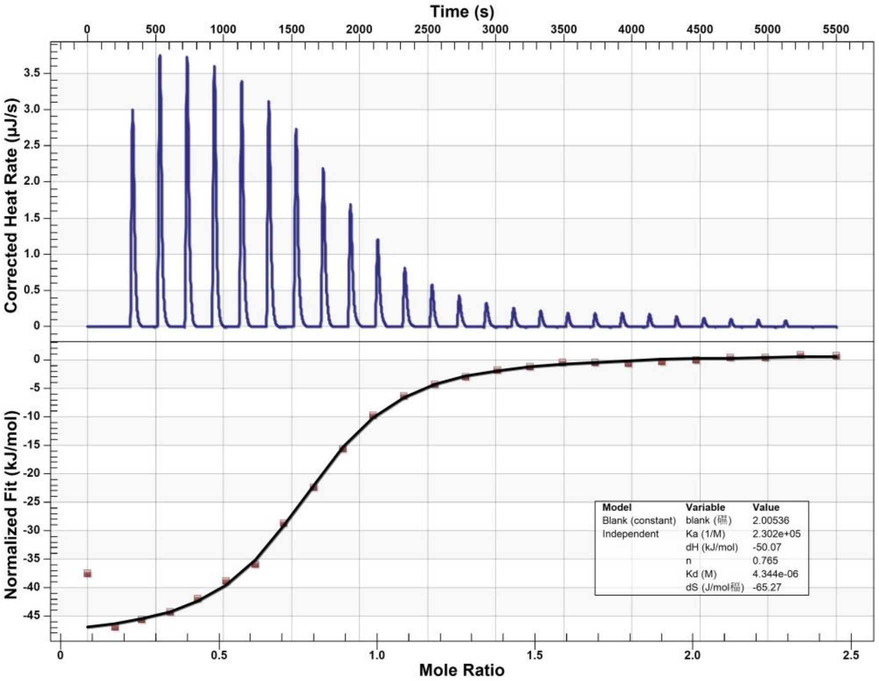

Figure S18: Titration plots (heat flow versus time and heat versus guest/host ratio) obtained from ITC experiments of **ZB4** with **13**-PF_6 in the 1:1 mixture of 1,2-dichloroethane and CH_3CN.

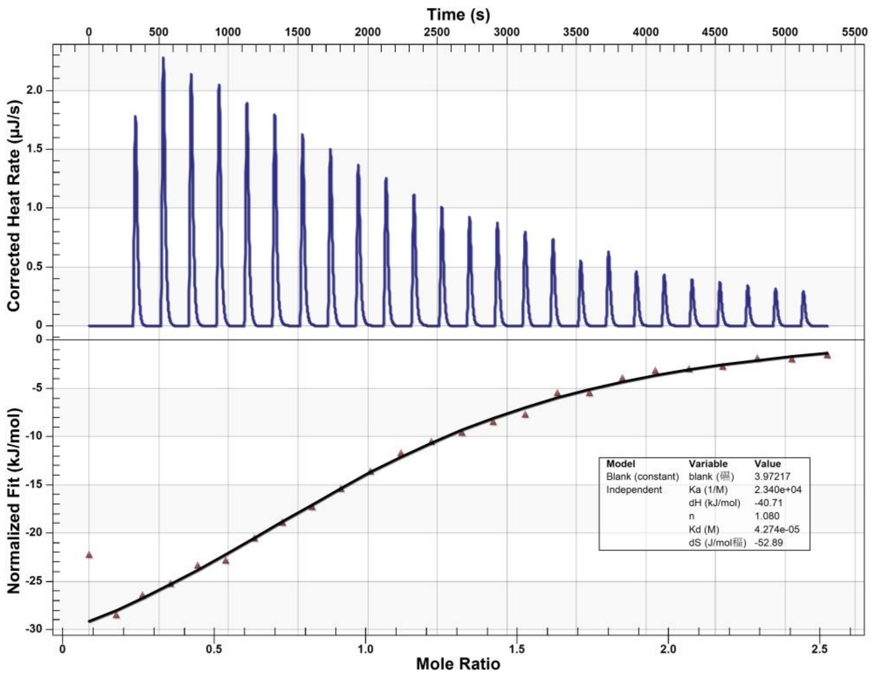

Figure S19: Titration plots (heat flow versus time and heat versus guest/host ratio) obtained from ITC experiments of **ZB4** with **14-**PF_6 in the 1:1 mixture of 1,2-dichloroethane and CH_3CN.

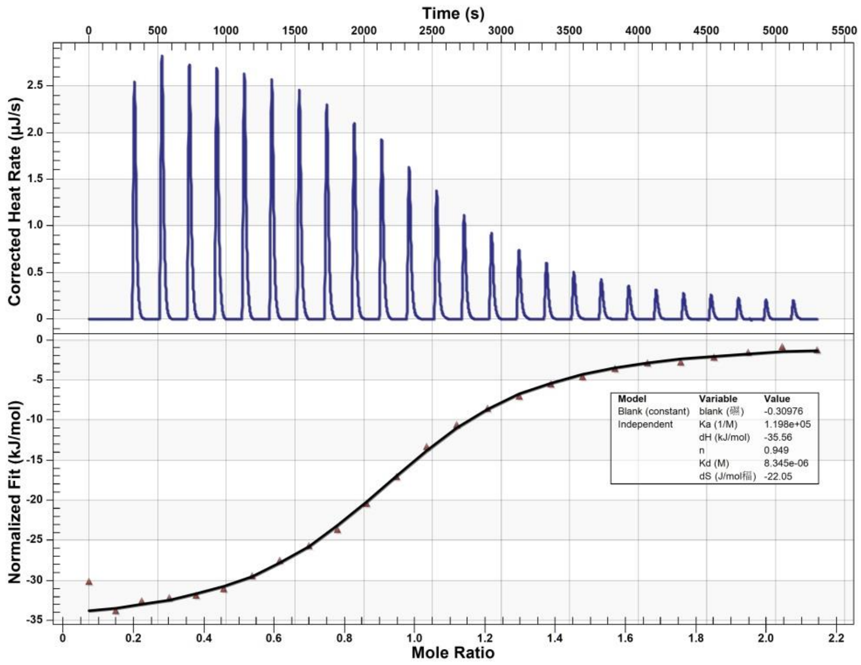

Figure S20: Titration plots (heat flow versus time and heat versus guest/host ratio) obtained from ITC experiments of **ZB4** with **15-**PF_6 in the 1:1 mixture of 1,2-dichloroethane and CH_3CN.

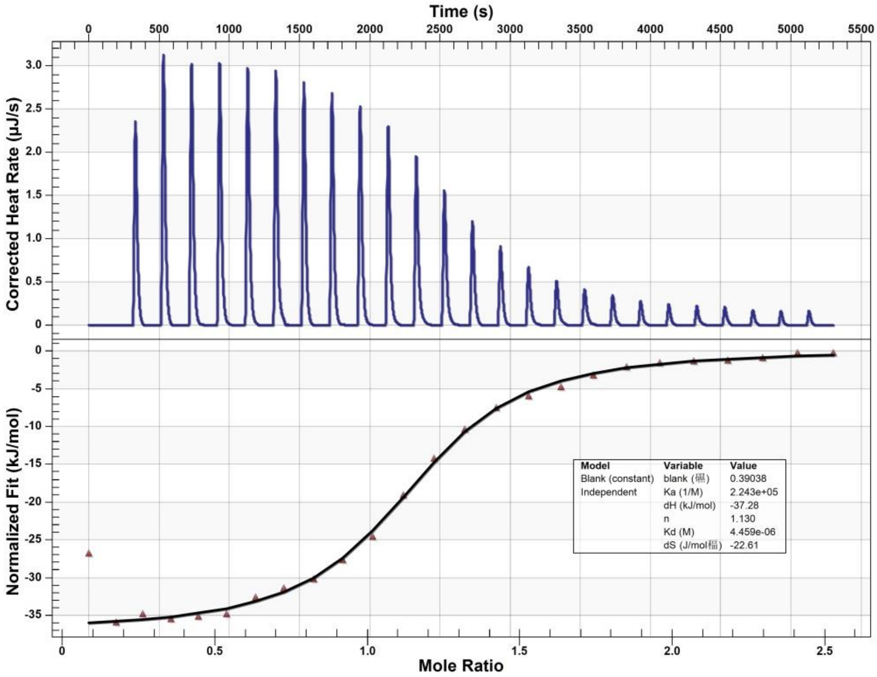

Figure S21: Titration plots (heat flow versus time and heat versus guest/host ratio) obtained from ITC experiments of **ZB4** with **16-**PF_6 in the 1:1 mixture of 1,2-dichloroethane and CH_3CN.

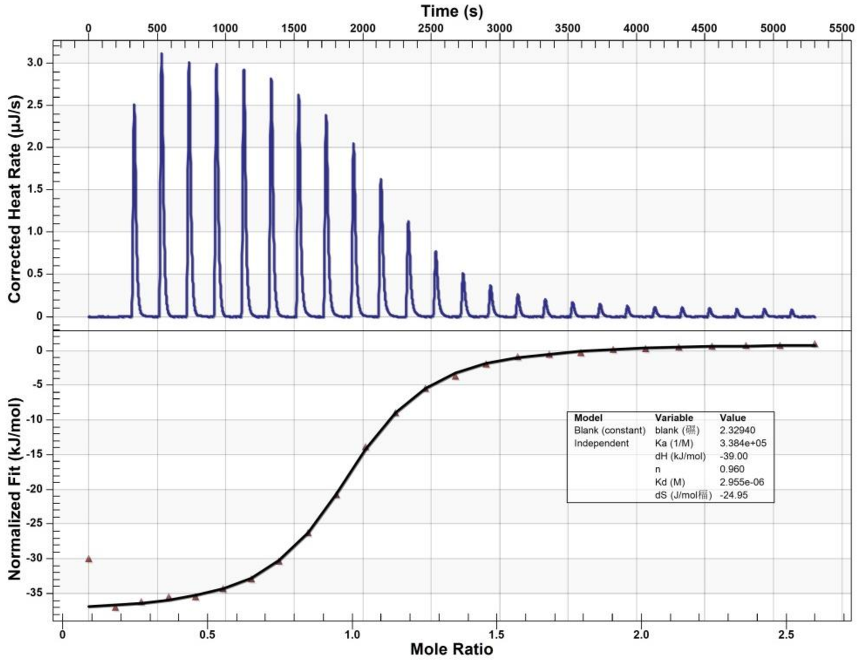

Figure S22: Titration plots (heat flow versus time and heat versus guest/host ratio) obtained from ITC experiments of **ZB4** with **17-**PF_6 in the 1:1 mixture of 1,2-dichloroethane and CH_3CN.

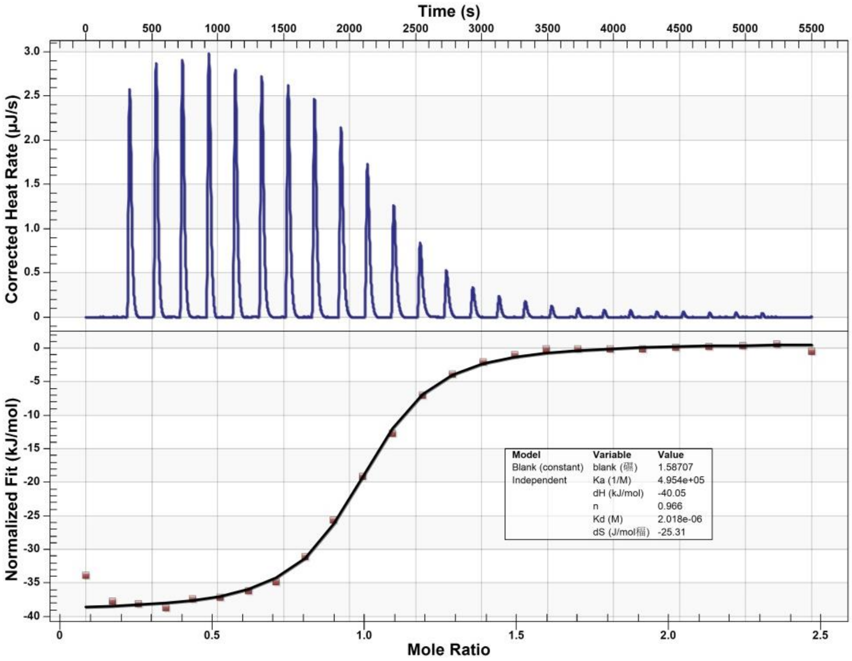

Figure S23: Titration plots (heat flow versus time and heat versus guest/host ratio) obtained from ITC experiments of **ZB4** with **18**-PF_6 in the 1:1 mixture of 1,2-dichloroethane and CH_3CN.

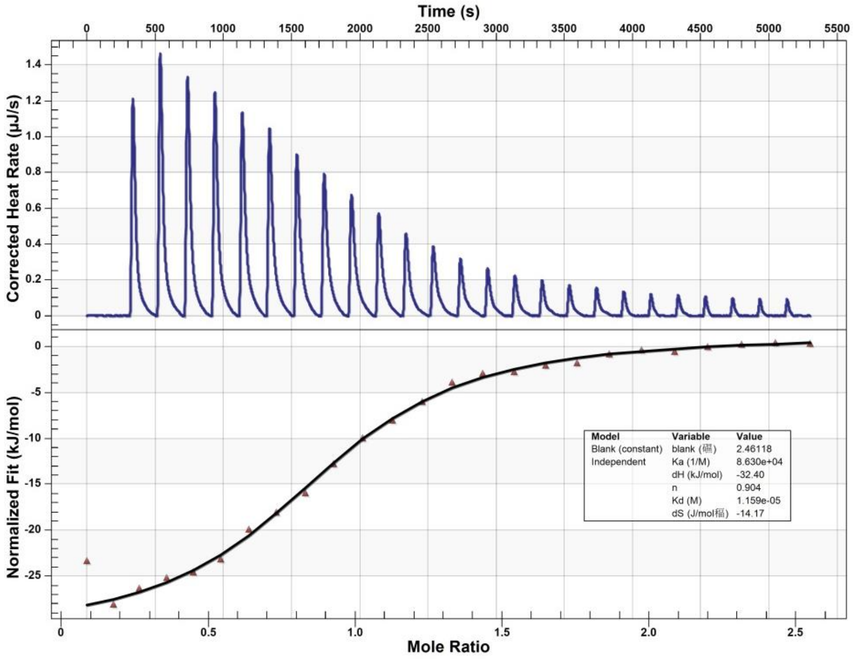

Figure S24: Titration plots (heat flow versus time and heat versus guest/host ratio) obtained from ITC experiments of **ZB4** with **19**-PF_6 in the 1:1 mixture of 1,2-dichloroethane and CH_3CN.

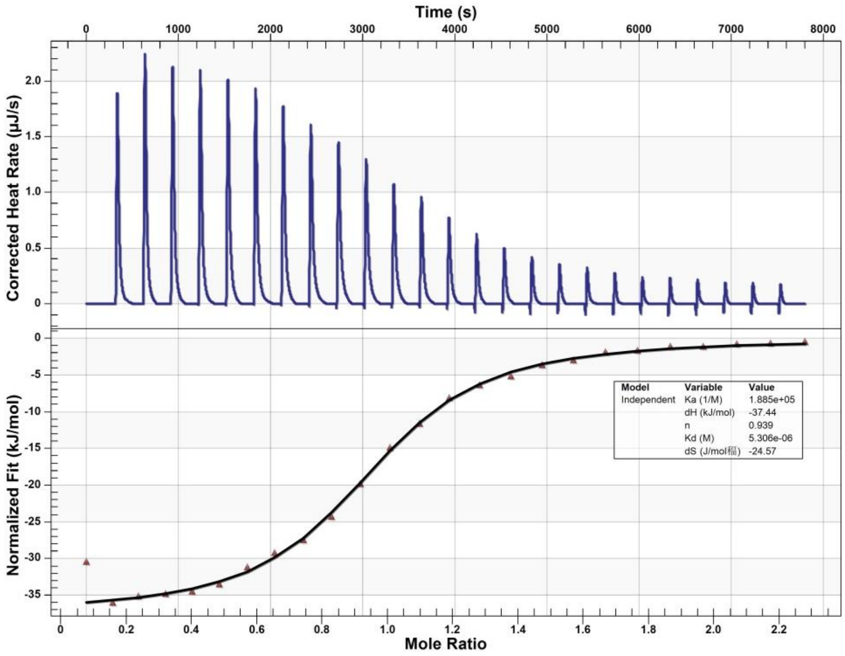

Figure S25: Titration plots (heat flow versus time and heat versus guest/host ratio) obtained from ITC experiments of **ZB4** with **20**-PF_6 in the 1:1 mixture of 1,2-dichloroethane and CH_3CN.

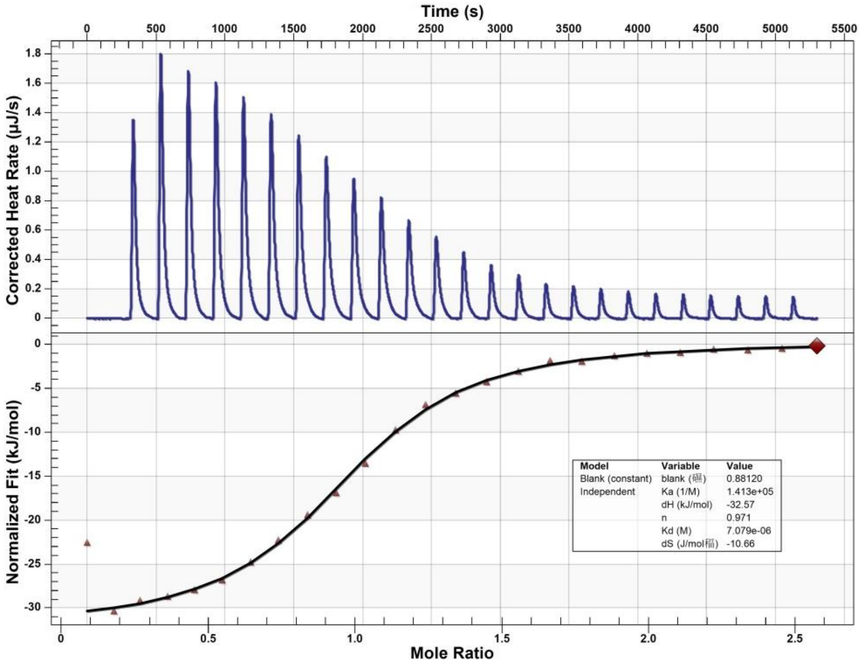

Figure S26: Titration plots (heat flow versus time and heat versus guest/host ratio) obtained from ITC experiments of **ZB4** with **21**-PF_6 in the 1:1 mixture of 1,2-dichloroethane and CH_3CN.

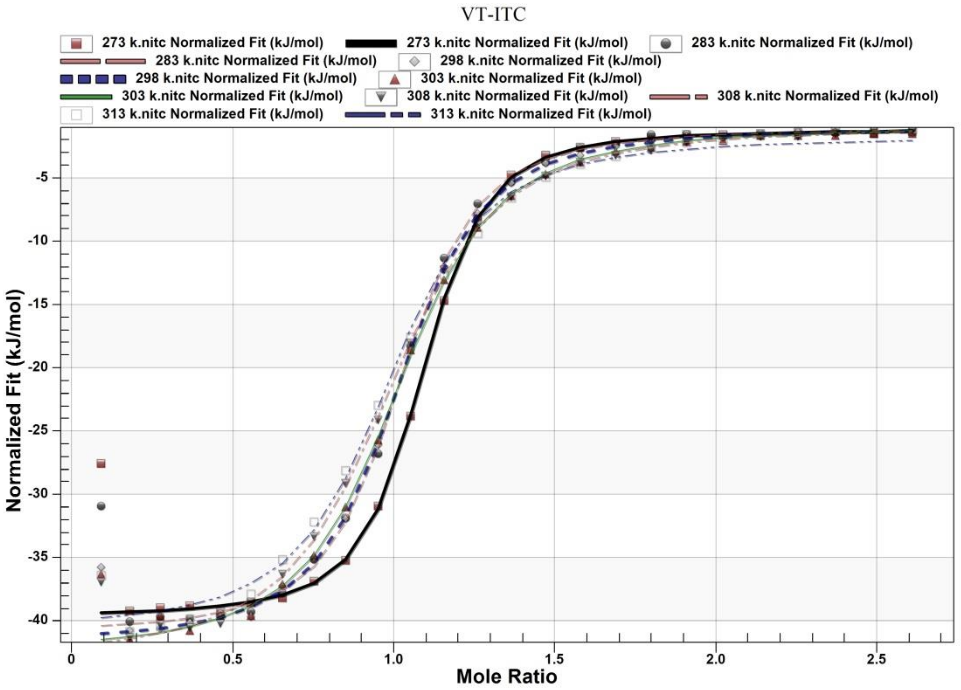

Figure S27: Titration plots (heat versus guest/host ratio) obtained from ITC experiments of **ZB4** with **18-**PF_6 in the 1:1 mixture of 1,2-dichloroethane and CH_3CN at different temperatures.

6. X-Ray single crystal data

Single crystals in this study were obtained by slow evaporation of MeCN (**ZB4**), of a 1:1 EtOAc/MeCN solution (**10**$^{+}$@**ZB4**), of a CH_2Cl_2 solution (**14**$^{+}$@**ZB4**) or of a 1:1 CH_2Cl_2/MeCN solution (**16^{+}@ZB4**, **18^{+}@ZB4**, **21**$^{+}$@**ZB4** and **18**$^{+}PF_6$). All these solutions (except for pure **ZB4** and **18**$^{+}PF_6$) contained 1:1 mixture of **ZB4** and corresponding guest **10**·PF_6, **14**·PF_6, **16**·PF_6, **18**·PF_6 or **21**·PF_6.

The reflections for **ZB4**, **10**$^{+}$@**ZB4**, **14**$^{+}$@**ZB4**, **18**$^{+}$@**ZB4**, **21**$^{+}$@**ZB4** and **18**$^{+}PF_6$ were collected at 123 K with an Agilent Super-Nova dual wavelength diffractometer using mirror-monochromatized CuKα (λ = 1.54184Å) radiation. The data for **16**$^{+}$@**ZB4** were collected at 293 K with a Rigaku Saturn724 CCD diffractometer using mirror-monochromatized MoKα (λ = 0.71073Å) radiation. *CrysAlisPro*[2] was used for both data collection and processing. The intensities were corrected for absorption using analytical face index absorption correction method[3] for all the data. The structures were solved by intrinsic phasing method with *SHELXT*[4] and refined by full-matrix least-squares methods using *SHELXL-2015* (or newer version).[5] All non-hydrogen atoms in the structures were refined with anisotropic thermal parameters. All hydrogen positions were refined using riding models.

The crystal of **ZB4** was found to be a two-component nonmerohedric twin and HKLF5 refinement was applied. In the crystal structure there are two crystallographically independent molecules in the asymmetric unit. The first molecule shows disorder in two butyl groups. The terminal propyl moiety of one disordered butyl group and the terminal ethyl moiety of the other disordered butyl group were refined by splitting over two parts with refined occupancies. In the second **ZB4** molecule only one butyl group shows significant disorder, in which the terminal propyl moiety was similarly splitted in two parts. Moderate geometric restraints were used to obtain chemically reasonable bond distances and angles, and stronger restraints (s = 0.01, st = 0.02) were applied to all atoms in disorders for reasonable ADPs. One additional geometric restraint was necessary to keep one disordered methylene hydrogen within reasonable distance from another methylene hydrogen of the same butyl group.

In the structure of **10**$^{+}$@**ZB4**, five of the eight butyl groups in the **ZB4** molecule were significantly disordered. In two of them all atoms of the butyl group were refined by splitting over two parts, two terminal ethyl moieties in the butyl groups were refined by splitting over two parts, and in the other one terminal propyl moiety was split in two parts with refined occupancies. Moderate geometric restraints were used to obtain chemically reasonable bond distances and angles, and stronger restraints (s = 0.01, st = 0.02) were applied to few atoms for reasonable anisotropic displacement parameters (ADPs). The guest **10**$^{+}$ also shows a 1:1 disorder, in which the carbon atoms were refined over two positions with fixed occupancies. Moderate geometrical restraints

were also applied for the guest molecule. The PF_6^- anion is also disordered in that way that four fluorine atoms were refined over two positions with fixed 50:50 occupancies. The geometry of the anion is moderately restrained and only a few stronger ADP restraints (s = 0.01, st = 0.02) were applied to phosphorus and one fluorine atom. One additional geometric restraint was necessary to keep one disordered methyl hydrogen within reasonable distance from methyl hydrogen of the adjacent **ZB4** molecule.

In the structure of **14**$^+$@**ZB4**, four of the eight butyl groups were found to be disordered. Two terminal propyl moieties of the butyl groups were refined by splitting over two positions with fixed occupancies, while only one methylene (second from oxygen atom) in two other butyl groups were split. Moderate geometric restraints were used to obtain chemically reasonable bond distances and angles, and stronger restraints (s = 0.01, st = 0.02) were applied to all atoms in disorders for reasonable ADPs. The ADPs of one terminal methyl carbon were further restrained very strongly (s = 0.005, st = 0.01). The cocrystallized CH_2Cl_2 molecule shows also disorder over two symmetrically equivalent positions and the ADPs of the other chlorine atom had to be restrained very strongly (s = 0.005, st = 0.01).

In **16**$^+$@**ZB4** four of the eight butyl groups in the **ZB4** molecule were disordered and all atoms of the butyl group were refined by splitting over two parts with fixed occupancies. Moderate geometric restraints were used to obtain chemically reasonable bond distances and angles. The geometry of disordered butyl groups were moderately and the ADPs strongly restrained (s = 0.01, st = 0.02).

The crystal of **18**$^+$@**ZB4** is a merohedral two-component twin and refined accordingly with BASF parameter of 0.105. Half of the eight butyl groups in **ZB4** show significant disorder. In two of them all atoms of the butyl group were refined by splitting over two parts, in one only the terminal ethyl moiety was split in two parts, and in other one only the terminal methyl moiety was split in two parts with refined occupancies. Moderate geometric restraints were used to obtain chemically reasonable bond distances and angles. The stronger restraints (s = 0.01, st = 0.02) were necessary to obtain reasonable ADPs for the carbon atoms. Also, similar ADP restraints were necessary for some carbon and oxygen atoms of **ZB4**.

In **21**$^+$@**ZB4,** five of the eight butyl groups in the **ZB4** molecule were disordered and then refined by splitting over two parts with fixed occupancies for the terminal one of two carbons based on the difference Fourier map. The geometry of disordered butyl groups were moderately and the ADPs strongly restrained (s = 0.01, st = 0.02). The O16 atom in the guest **21**$^+$ was also badly disordered. Its ADP was restrained very strongly (s = 0.005, st = 0.01). In addition, SQUEEZE[6] was used to treat the data due to the highly disordered solvent molecules (MeCN + CH_2Cl_2).

In the crystal structure of **18**$^+$$PF_6$ there is only a half molecule in the symmetric unit. It aimlessly crystallized out from one of the samples containing also **ZB4** host (in 1:1 ratio). Its crystal structure is previously unknown, to the best of our knowledge, and is thus presented here.

The details of the data collection and refinement results are summarized as follows.

Crystal data **ZB4**: 0.18×0.07×0.06 mm, $C_{80}H_{104}O_{12}$, M = 1257.63, triclinic, P-1, a = 14.1631(5) Å, b = 22.7817(8) Å, c = 23.1745(3) Å, α = 90.116(3)°, β = 106.804(3)°, γ = 91.110(3)°, V = 7156.6(4) Å^3, Z = 4 (Z' = 2), ρ = 1.167 g cm^{-3}, μ = 0.610 mm^{-1}, F(000) = 2720, 25179 reflections (θ_{max} = 67.000°) measured (25179 unique, R_{int} = 0.0657 (before HKLF5 refinement), completeness = 99.0%), Final R indices ($I > 2\sigma(I)$): R_1= 0.1287, wR_2 = 0.3851, R indices (all data): R_1= 0.1508, wR_2 = 0.4015. GOF = 1.081 for 1733 parameters and 241 restraints, largest diff. peak and hole 0.563 / –0.566 eÅ^{-3}. CCDC-1838266 contains the supplementary data for this structure.

Crystal data **10**$^+$@**ZB4**: 0.25×0.20×0.10 mm, $C_{90}H_{114}CoF_6O_{12}P$, M = 1591.71, triclinic, P-1, a = 13.0173(3) Å, b = 13.1783(3) Å, c = 24.8011(5) Å, α = 90.983(2)°, β = 100.376(2) °, γ = 90.711(2)°, V = 4182.66(16) Å^3, Z = 2, ρ = 1.264 g cm^{-3}, μ = 2.395 mm^{-1}, F(000) = 1692, 28280 reflections (θ_{max} = 67.684°) measured (16379 unique, R_{int} = 0.0243, completeness = 99.4%), Final R indices ($I > 2\sigma(I)$): R_1= 0.0516, wR_2 = 0.1425, R indices (all data): R_1= 0.0567, wR_2 = 0.1477. GOF = 1.029 for 1212 parameters and 372 restraints, largest diff. peak and hole 0.607 / –0.529 eÅ^{-3}. CCDC-1838267 contains the supplementary data for this structure.

Crystal data **14**$^+$@**ZB4**: 0.17×0.15×0.04 mm, $C_{105}H_{142}Cl_2F_6NO_{12}P$, M = 1826.06, orthorhombic, $Pbcn$, a = 31.3606(13) Å, b = 18.8882(3) Å, c = 17.0957(3) Å, $\alpha = \beta = \gamma$ = 90°, V = 10126.6(5) Å^3, Z = 4 (Z' = 0.5), ρ = 1.198 g cm^{-3}, μ = 1.292 mm^{-1}, F(000) = 3912, 22798 reflections (θ_{max} = 67.684°) measured (9257 unique, R_{int} = 0.0240, completeness = 99.4%), Final R indices ($I > 2\sigma(I)$): R_1= 0.0866, wR_2 = 0.2557, R indices (all data): R_1= 0.1020, wR_2 = 0.2752. GOF = 1.033 for 626 parameters and 107 restraints, largest diff. peak and hole 0.889 / –0.784 eÅ^{-3}. CCDC-1838268 contains the supplementary data for this structure.

Crystal data **16**$^+$@**ZB4**: 0.30×0.22×0.15 mm, $C_{96}H_{122}Cl_2F_6O_{12}P$, M = 1697.81, orthorhombic, space group $Pbcn$, a = 13.342(2) Å, b = 22.151(3) Å, c = 31.744(5) Å, α = 90°, β = 90°, γ = 90°, V = 9381(3) Å^3, Z = 4 (Z' = 0.5), ρ = 1.202 g cm^{-3}, μ = 0.156 mm^{-1}, F(000) = 3616, 95417 reflections (θ_{max} = 49.998°) measured (8243 unique, R_{int} = 0.0368, completeness = 99.8%), Final R indices ($I > 2\sigma(I)$): R_1= 0.1014, wR_2 = 0.2880, R indices (all data): R_1= 0.1062, wR_2 = 0.2927. GOF = 1.085 for 596 parameters and 2 restraints, largest diff. peak and hole 1.29 /–0.47 eÅ^{-3}. CCDC-1836247 contains the supplementary data for this structure.

Crystal data **18**$^+$@**ZB4**: 0.12×0.08×0.03 mm, $C_{96}H_{122}F_6I_2NO_{12}P$, M = 1880.71, monoclinic, $C2/c$, a = 18.9755(4) Å, b = 17.0235(4) Å, c = 56.3421(12) Å, $\alpha = \gamma$ =

90°, β = 90.795(2)°, V = 18198.4(7) Å^3, Z = 8, ρ = 1.373 g cm^{-3}, μ = 6.190 mm^{-1}, F(000) = 7808, 29903 reflections (θ_{max} = 67.275°) measured (15652 unique, R_{int} = 0.0644, completeness = 97.9%), Final R indices ($I > 2\sigma(I)$): R_1= 0.1398, wR_2 = 0.2997, R indices (all data): R_1= 0.1552, wR_2 = 0.3098. GOF = 1.057 for 1167 parameters and 478 restraints, largest diff. peak and hole 3.324 (1.02 Å from I1) / –2.672 (0.91 Å from I1) eÅ^{-3}. CCDC-1838269 contains the supplementary data for this structure.

Crystal data **21**$^+$@**ZB4**: 0.06×0.14×0.24 mm, $C_{96}H_{122}N_4O_{16}PF_6$ M = 1718.93, triclinic, space group P-1, a = 12.6912(5) Å, b = 12.6941(4) Å, c = 32.8983(14) Å, α = 80.846(3)°, β = 85.532(4)°, γ = 84.359(3)°, V = 5196.7(4) Å^3, Z = 2, ρ = 1.099 g cm^{-3}, μ = 0.807 mm^{-1}, F(000) = 1832, 30230 reflections (θ_{max} = 66.749°) measured (18207 unique, R_{int} = 0.0481, completeness = 98.95%), Final R indices ($I > 2\sigma(I)$): R_1= 0.1046, wR_2 = 0.2910, R indices (all data): R_1= 0.1309, wR_2 = 0.3169. GOF = 1.035 for 1189 parameters and 148 restraints, largest diff. peak and hole 1.014 /–0.512 eÅ^{-3}. CCDC- 1836509 contains the supplementary data for this structure.

Crystal data **18**$^+$$PF_6$: 0.20×0.16×0.11 mm, $C_{16}H_{18}F_6I_2NP$, M = 623.08, orthorhombic, $Pnma$, a = 9.9737(2) Å, b = 8.5642(2) Å, c = 23.0722(4) Å, $\alpha = \beta = \gamma$ = 90°, V = 1970.75(7) Å^3, Z = 4 (Z' = 0.5), ρ = 2.100 g cm^{-3}, μ = 26.364 mm^{-1}, F(000) = 1184, 12149 reflections (θ_{max} = 67.684°) measured (2132 unique, R_{int} = 0.0609, completeness = 99.9%), Final R indices ($I > 2\sigma(I)$): R_1= 0.0295, wR_2 = 0.0728, R indices (all data): R_1= 0.0311, wR_2 = 0.0749. GOF = 1.056 for 136 parameters and 0 restraints, largest diff. peak and hole 0.653 / –2.116 (0.81 Å from I2) eÅ^{-3}. CCDC-1838270 contains the supplementary data for this structure.

7. References

[1] Huang, G.; He, Z.; Cai, C.-X.; Pan, F.; Yang, D.; Rissanen K.; Jiang. W. *Chem. Commun.* **2015**, *51*, 15490-15493.

[2] *CrysAlisPro* **2014**, Rigaku Oxford Diffraction. Version 1.171.37.35 (or newer).

[3] R. C. Clark, J. S. Reid, *Acta Crystallogr., Sect. A: Found. Crystallogr.* **1995**, *51*, 887.

[4] G. M. Sheldrick, *Acta Crystallogr., Sect. A: Found. Adv.* **2015**, *71*, 3-8.

[5] G. M. Sheldrick, *Acta Crystallogr., Sect. C: Struct. Chem.* **2015**, *71*, 3-8.

[6] A. L. Spek, *Acta Crystallogr., Sect. C: Struct. Chem.* **2015**, *71*, 9-18.
